# An observational study investigating soluble immune checkpoints as indicators of severe COVID-19

**DOI:** 10.1128/spectrum.03776-23

**Published:** 2024-05-29

**Authors:** Ricarda Cortés-Vieyra, Sergio Gutiérrez-Castellanos, Anel Gómez-García, Alejandro Bravo-Patiño, Fernando Calderón-Rico, José Daniel Martínez-Sepúlveda, Roberto Ortega-Flores, Francisco Perez-Duran, Luis Enrique Franco-Correa, Alicia Gabriela Zamora-Avilés, Rosa Elvira Nuñez-Anita

**Affiliations:** 1 Facultad de Medicina Veterinaria y Zootecnia (FMVZ), Universidad Michoacana de San Nicolás de San Nicolás de Hidalgo (UMSNH), Morelia-Zinapécuaro, Mexico; 2 Centro de Investigación Biomédica de Michoacán, División de Investigación Clínica, Instituto Mexicano del Seguro Social, Morelia, Mexico; 3 Centro Multidisciplinario de Estudios en Biotecnología de la FMVZ, UMSNH, Morelia-Zinapécuaro, Mexico; 4 Instituto de investigaciones Químico Biológicas, UMSNH, Morelia, México; Universidade Federal do Rio de Janeiro, Rio de Janeiro, Brazil

**Keywords:** COVID-19, immune checkpoints, SARS-CoV-2, observational study

## Abstract

This study aimed to investigate the immunomodulatory behavior of soluble immune checkpoints (sICPs) and other biomarkers in the pathophysiology of SARS-CoV-2 infection. The study included 59 adult participants, 43 of whom tested positive for SARS-CoV-2. Patients were divided into three cohorts: those with moderate disease (*n* = 16), recovered patients with severe disease (*n* = 13), and deceased patients with severe disease (*n* = 16). In addition, 16 participants were pre-pandemic subjects negative for SARS-CoV-2. The relative activity of neutralizing antibodies (rNAbs) against SARS-CoV-2 and the values of 14 sICPs in peripheral blood were compared between the four groups. Because the increase of markers values of inflammation [NLR > 12; CRP > 150 mg/L] and venous thromboembolism [D-dimer > 0.5 mg/L] has been associated with mortality from COVID-19, the total and differential leukocyte counts, the NLR, and CRP and D-dimer values were obtained in patients with severe disease. No differences in rNAbs were observed between the cohorts. Only the levels of five sICPs, sCD27, sHVEM sTIM-3, sPD-1, and sPDL-1, were significantly higher in patients with severe rather than moderate disease. The sPDL-2 level and NLR were higher in deceased patients than in recovered patients. However, there was no difference in CRP and D-dimer values between the two groups. Of the five soluble biomarkers compared among patients with severe disease, only sPDL-2 was higher in deceased patients than in recovered patients. This suggests that immuno-inhibitory sICPs might be used as indicators for severe COVID-19, with sPDL-2 used to assess individual risk for fatality.

**IMPORTANCE:**

COVID-19, the disease caused by a SARS-CoV-2 infection, generates a broad spectrum of clinical symptoms, progressing to multiorgan failure in the most severe cases. As activation of the immune system is pivotal to eradicating the virus, future research should focus on identifying reliable biomarkers to efficiently predict the outcome in severe COVID-19 cases. Soluble immune checkpoints represent the function of the immune system and are easily determined in peripheral blood. This research could lead to implementing more effective severity biomarkers for COVID-19, which could increase patients’ survival rate and quality of life.

## INTRODUCTION

In countries with a successful vaccination campaign against severe acute respiratory syndrome coronavirus 2 (SARS-CoV-2) leading to COVID-19, immunity in most of the population has been accomplished. However, older adults with decompensation of chronic comorbidities represent most patients admitted to hospitals with a positive SARS-CoV-2 test and severe complications, regardless of vaccination status (1). Moreover, infection with SARS-CoV-2 with antigenic escape mutations can reduce the efficacy of vaccines (2). According to the World Health Organization (3), COVID-19 is currently an endemic disease, implying that vulnerable persons may become a persistent medical issue; therefore, strategies to combat severe COVID-19 are essential.

Immune checkpoints (ICPs) are co-stimulatory and co-inhibitory molecules that allow full lymphocyte activation or act as lymphocyte regulators that avoid excessive responses through inhibiting and activating pathways initiated by ligand-receptor interactions (4). Co-inhibitory ICPs are crucial for maintaining self-tolerance and modulating the duration and amplitude of physiological immune responses in peripheral tissues, protecting them from damage during the eradication of infections and malignancies and providing resistance against auto-antigens (5, 6).

In the context of cancer, blocking ICPs on the cell membrane with monoclonal antibodies is a successful treatment strategy, but its cost is prohibitive (7). ICPs also exist in soluble forms (sICPs) that can diffuse in serum and, similar to the membrane-bound form, modulate immune cell functions by stimulating and inhibiting diverse pathways (8). However, the production, regulation, and biological significance of sICPs in health and disease remain elusive. Hence, there is an urgent need to study the role of sICPs in cancer (7) and infectious diseases with global repercussions such as COVID-19 (9).

A clinical study in China found an association between a simultaneous and persistent increase of 11 sICPs (primarily those with immuno-inhibitory functions) and disease severity during hospitalization (10). Furthermore, they reported that increases of soluble indoleamine 2,3-dioxygenase 1 (sIDO), sCD137 (also known as s4-1BB), soluble T-cell immunoglobulin domain and mucin domain 3 (sTIM3), and sCD27 had good predictive value for COVID-19 severity and the inflammatory cytokines, IL-6 IL-10, IP-10, and IL-18. Another clinical study in Korea reported that increased levels of sCD27, sCD40, sCD152, soluble cytotoxic T-lymphocyte-associated protein 4 (sCTLA-4), and sTIM3 were significantly higher in COVID-19 patients who died than in survivors (11). Moreover, in independent studies that included COVID-19 patients with severe to critical disease, increased levels of specific sICPs, such as the non-classic histocompatibility antigen G (HLA-G), sCD279, soluble programmed cell death 1 (sPD-1), soluble programmed cell death-ligand 1 (sPDL1), sTIM-3, and soluble CD137 (sCD137) (12–18) were found in peripheral blood. However, to determine whether sICPs can function as potential blood biomarkers and therapeutic targets in COVID-19, more studies are necessary to better understand their immunomodulatory behavior in the pathophysiology of this disease.

We attempted to identify soluble biomarkers easily determinable in serum from COVID-19 patients and to confirm the behavior of sICPs by SARS-CoV-2 infection in a different scenario than those previously described, through the inclusion of a COVID-19 vaccinated cohort (with moderate disease) and two partially vaccinated cohorts infected with the Delta variant, which is associated with high viral loads and disease severity/mortality (19). Hence, we investigated the relative activity of neutralizing antibodies (rNAbs) against SARS-CoV-2 and simultaneously analyzed five co-stimulatory sICPs: sCD28, s4-1BB; soluble tumor necrosis factor receptor superfamily member 9 (sTNFRSF9); soluble glucocorticoid tumor necrosis factor receptor family-related receptor (sGITR); and sCD27 along with the soluble herpes virus entry mediator (sHVEM). Nine co-inhibitory sICPs were also analyzed: sCD80, CD152, sCTLA4, (sIDO), soluble B and T lymphocyte attenuator (sBTLA), soluble lymphocyte-activation gene 3 (sLAG3), sTIM3, sPD-1, sCD273/sPDL-1 as well as the sPD-1-ligand 2 (sCD274/sPDL-2)—the latter with a recovery or death outcome. Additionally, total and differential leukocyte counts, the neutrophil to lymphocyte ratio (NLR), and C-reactive protein (CRP) and D-dimer values were determined in patients with severe COVID-19.

## RESULTS

### Demographic and clinical characteristics of COVID-19 patients

**Fig 1:**
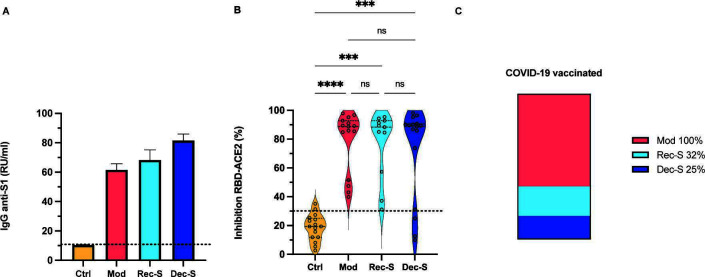
Seroconversion and relative neutralization activity against SARS-CoV-2. (**A**) Qualitative detection of IgG antibodies against the SARS-CoV-2 S protein. Values greater than 11 RU/mL (dashed line) are considered a positive seroconversion against SARS-CoV-2. (**B**) Relative neutralization activity against SARS-CoV-2. A percentage of the inhibition of RBD-ACE2 interaction above 30% (dashed line) was interpreted as positive neutralizing antibody activity. The center horizontal lines of the violin plots represent the median, and the horizontal lines at the ends represent the lower and upper quartiles. (**C**) Percentage of vaccinated patients against COVID-19. The *P*-values were obtained by analyzing the variables with the Kruskal-Wallis test with Dunnett’s multiple comparisons. ***P ≤ 0.0004; ****P < 0.0001; ns, not significant. Ctrl (negative controls); Mod (patients with moderate disease); Rec-S (recovered patients with severe disease); Dec-S (deceased patients with severe disease).

Patients with moderate disease had a lower mean age than those with severe disease. There was no significant gender difference between the groups although there were more male patients with severe disease (Table 1). Both vaccinated and unvaccinated patients against COVID-19 were included in the clinical study. Vaccinated individuals received BioNTech-Pfizer, Oxford-AstraZeneca, or Cansino Biologics Ad5-nCoV-S formulation schemes. All patients with moderate disease were vaccinated against COVID-19 with a two-dose schedule. In contrast, nearly three-quarters of patients with severe disease had not received a dose of any COVID-19 vaccine (Table 1; Fig. 1).

**TABLE 1:** Patient baseline demographics and disease characteristics^*c*^^,*d*^

Characteristic	Negative controls^*a*^ (*n* = 16)		Patients with moderate disease (*n* = 16)		Recovered with severe disease (*n* = 13)		Deceased with severe disease (*n* = 16)		*P*
Age, years mean (range)	–	–	43*	(24–62)	60	(20–82)	69	(42–88)	**<0.0001^*b*^**
	N	%	N	%	N	%	N	%	
Male sex	–	–	6/16	37	9/13	69	8/16	50	0.2343
COVID-19 vaccinated	–	–	16*	100	4	32	4	25	**<0.0001**
Comorbidities									
COPD	**–**	–	2	13	2	15	4	25	0.6291
Diabetes mellitus	–	–	1	6	4	31	7	44	0.0521
Asthma	–	–	0	0	1	8	0	0	0.2840
Immunosuppression	–	–	0	0	1	8	0	0	0.2840
Smoking	–	–	2	13	2	15	1	6	0.7209
Obesity	–	–	3	19	3	23	2	13	0.7539
Hypertension	–	–	1	1	6	46	12*	75	**0.0004**
Cardiovascular disease	–	–	0	0	3	23	1	6	0.0850
Renal disease	–	–	0	0	0	0	1	6	0.3958
COVID-19 symptoms									
Sudden onset	–	–	1	6	0	0	1	6	0.6537
Fever	–	–	7	44	9	69	11	69	0.2551
Dry cough	–	–	10	63	8	62	15	94	0.0708
Headache	–	–	12	75	6*	46	14	88	**0.0462**
Odynophagia	–	–	11	69	7	54	5	31	0.1024
Tiredness	–	–	8*	50	1	8	3	19	**0.0252**
Myalgia	–	–	9	56	6	46	9	56	0.8275
Arthralgia	–	–	8	50	7	54	9	56	0.9382
Prostration	–	–	2	13	0	0	0	0	0.1500
Rhinorrhea	–	–	3	19	3	23	1	6	0.4192
Chills	–	–	6*	38	1	8	1	6	**0.0366**
Dyspnea	–	–	1*	6	10	77	15	94	**<0.0001**
Chest pain	–	–	1*	6	8	62	4	25	**0.0044**
Anosmia	–	–	0	0	1	8	0	0	0.2840
Dysgeusia	–	–	1	6	1	8	0	0	0.5516
Influenza-like illness	–	–	14*	88	3	23	1	6	**<0.0001**
Severe, acute respiratory infection	–	–	–	–	10	77	15	94	0.2994
Radiographic pneumonia	–	–	0	0	2	15	7*	44	**0.0074**
Days hospitalized									
2–7	–	–	–	–	7	54	3	19	0.0641
8–14	–	–	–	–	2	15	7	44	0.1296
15–21	–	–	–	–	3	23	4	25	>0.9999
22–26	–	–	–	–	1	8	1	6	>0.9999
27–34 days	–	–	–	–	0	0	1	6	>0.9999
Days between onset of clinical symptoms and obtaining a blood sample									
2–7	–	–	8	50	7	54	3	19	0.0946
8–14	–	–	5	31	2	15	7	44	0.2602
15–21	–	–	1	6	3	23	3	19	0.4192
22–28	–	–	2	13	1	8	3	18	0.6791

^*a*^ Blood samples from healthy individuals (pre-pandemic) were used as negative controls.

^*b*^ The *P-*value for age was analyzed using the Kruskal-Wallis test.

^*c*^ " * " indicates the data for which the significant p-value was observed.

^*d*^ COPD, chronic, obstructive pulmonary disease.

Seventy-five percent of the deceased patients had hypertension. While several patients with severe COVID-19 had diabetes, the difference was not statistically different from those with moderate symptoms. No significant differences between patient groups were recorded for the other comorbidities (Table 1). None of the patients reported having tuberculosis, cancer, or HIV.

Most of the patients with SARS-CoV-2 infection presented with headache. In addition, patients with moderate disease complained of tiredness and chills. The most common symptoms in patients with severe disease were dyspnea and chest pain, and they complained of an influenza-like illness. The majority of patients with severe disease had severe acute respiratory infections. None of the patients with moderate disease presented with radiographic pneumonia, which was present in approximately half of the severely ill patients who died (Table 1). Moreover, none of the patients with moderate disease were hospitalized. Although all patients with severe disease were hospitalized, half of the recovered patients remained hospitalized for 7 days or fewer, while most of the deceased patients were hospitalized between 8 and 21 days (Table 1). Blood samples from patients with moderate and severe disease who recovered were primarily collected within the first 14 days of clinical symptom onset. For the deceased patients, most samples were collected between 8 and 14 days after symptom onset (Table 1).

### Relative activity of neutralizing antibodies against SARS-CoV-2

Before determining the rNAbs against SARS-CoV-2, seroconversion to SARS-CoV-2 infection was established by detecting IgG antibodies against the S1 subunit of the virus. Seronegativity was confirmed in control sera, and seroconversion was confirmed in patients positive for SARS-CoV-2 infection (Fig. 1A). The three cohorts of COVID-19 patients showed a significantly higher percentage of receptor-binding domain (RBD) to angiotensin-converting enzyme-2 (ACE2) interaction inhibition (rNAbs) than the control group (Fig. 1B). However, the group of deceased patients (of whom 25% were vaccinated) showed a lower trend for the percentage of RBD-ACE2 inhibition compared to patients with moderate disease (100% vaccinated). There was no statistically significant difference in the percentage of RBD-ACE2 inhibition between the group of recovered patients (32% vaccinated) and the other COVID-19 cohorts (Fig. 1B and C).

### Comparison of soluble immune checkpoints in COVID-19 patient cohorts

#### Co-stimulatory soluble immune checkpoints in COVID-19 patients

**Fig 2:**
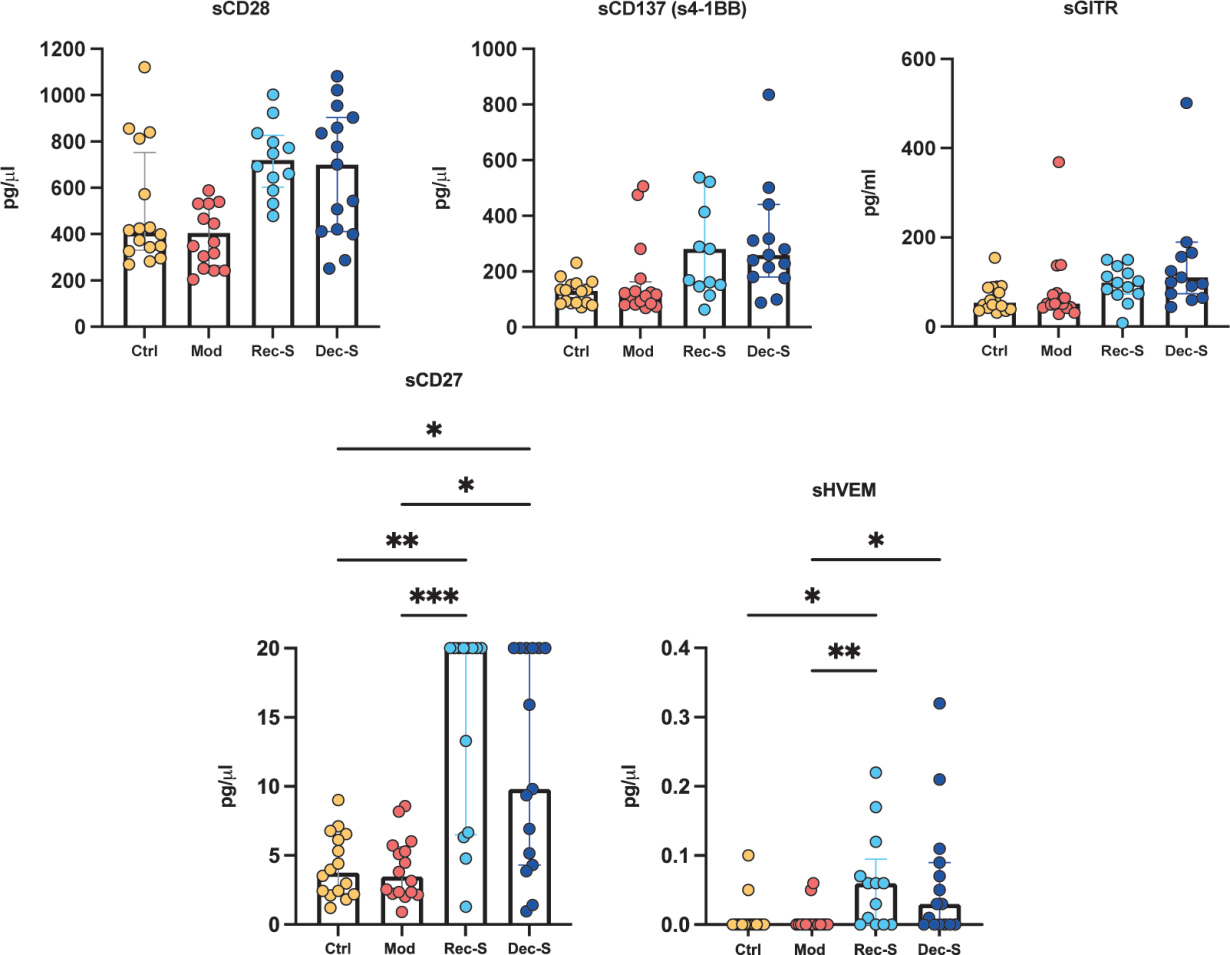
Stimulatory soluble immune checkpoints in COVID-19 patients. Medians are plotted with the interquartile ranges. The *P*-values were obtained by analyzing the variables with the Kruskal-Wallis test with Dunnett’s multiple comparison. *P ≤ 0.0417; **P ≤ 0.0097; *** *P* = 0.0009.

The median levels of the co-stimulatory sICPs (sCD28, s41BB, and sGITR) in sera showed no difference between the four cohorts. However, the median levels of sCD27 were significantly higher in recovered and deceased patients with severe disease compared to the control group and both when compared to moderate. Likewise, the median level of sHVEM was significantly higher in recovered patients with severe disease compared to the control group and patients with moderate disease and was significantly higher in deceased patients compared to those with moderate disease (Fig. 2).

#### Co-inhibitory soluble immune checkpoints in COVID-19 patients

**Fig 3:**
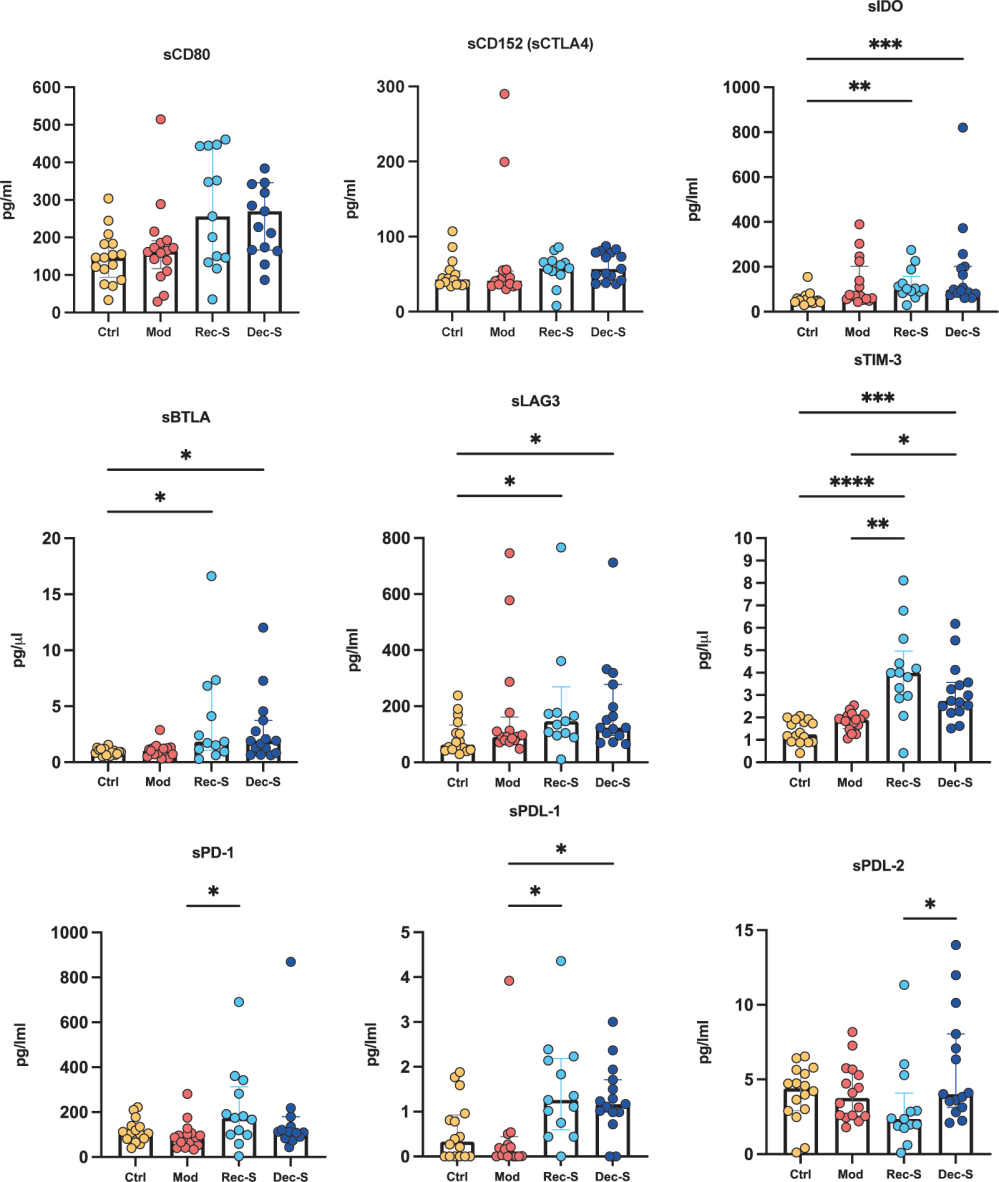
Inhibitory soluble immune checkpoints in COVID-19 patients. Medians were plotted with interquartile ranges. The *P*-values were obtained by analyzing the variables with the Kruskal-Wallis test with Dunnett’s multiple comparison. *P ≤ 0.0470; **P ≤ 0.0029; *** *P* ≤ 0.0003; ***P < 0.0001.

There was no significant difference in the median levels of sCD80 and sCTLA4 between the cohorts. While the median levels of sIDO, sBTLA, and sLAG3 were significantly higher in recovered and deceased patients with severe disease than the control group, there was no difference when compared with patients with moderate disease. Levels of sTIM3 were significantly higher in recovered and deceased patients compared with the control and moderate disease groups. Levels of sPD-1 and its ligand sPDL1 were significantly higher in recovered patients than in those with moderate disease. sPDL-1 also was higher in deceased patients than in those with moderate COVID-19. Notably, only sPDL-2 levels significantly differed between recovered and deceased patients with severe disease, with the median serum level higher in deceased patients (Fig. 3).

### Hematological data of severe COVID-19 patients

**Fig 4:**
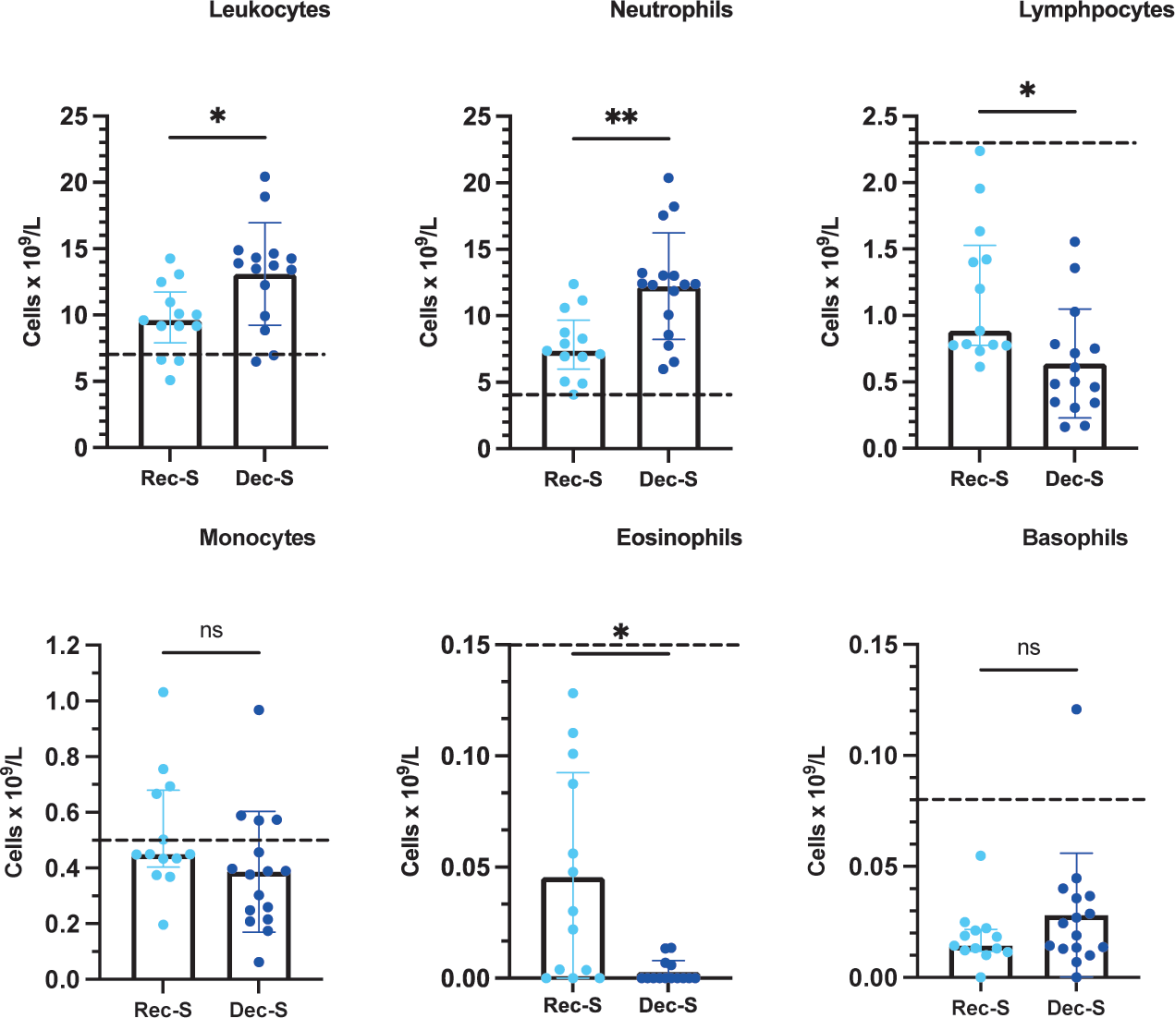
Total and differential leukocyte counts in COVID-19 patients with severe disease. Medians are plotted with interquartile ranges. Analyses were performed using a paired Wilcoxon signed-rank test. The dashed lines indicate the means of healthy subjects (20). Forty-two WBC differential counts from Rec-S and 30 WBC differential counts from Dec-S *P ≤ 0.0117; **P = 0.0077; ns *P* > 0.0500.

**Fig 5:**
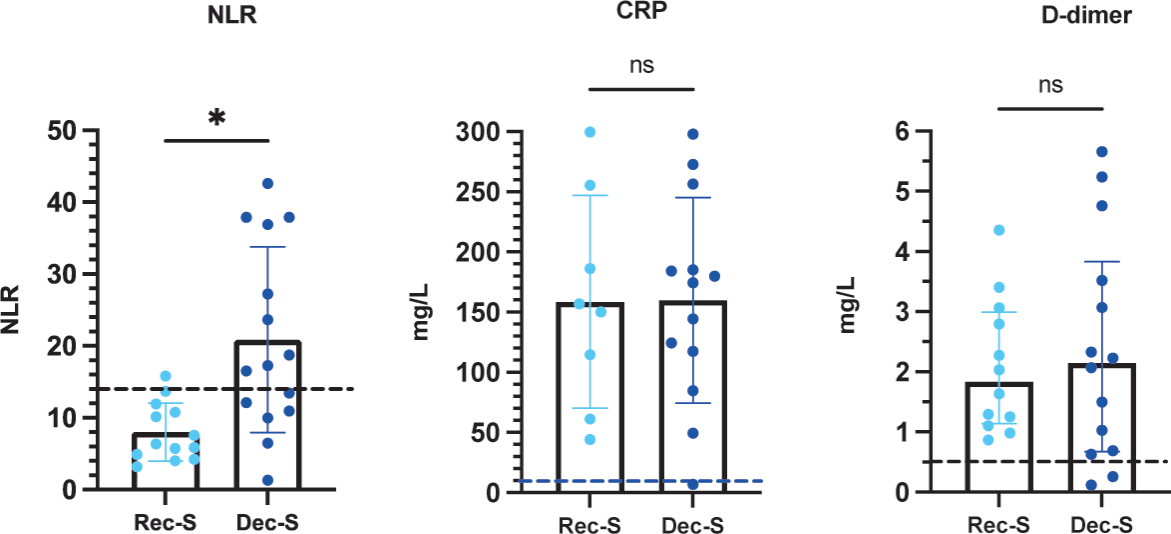
Levels of acute inflammation and venous thromboembolism markers in COVID-19 patients with severe disease. Means and standard errors were plotted for parametric data (NLR and CRP). For D-dimers, medians were plotted with interquartile ranges. Analyses were performed using a paired *t*-test (NLR: 42 determinations from Rec-S and 30 from Rec-D and CRP: 18 determinations from Rec-S and 32 from Rec-D) or paired Wilcoxon signed-rank test (D-dimer: 58 determinations from Rec-S and 56 from Rec-D). The dashed lines indicate the NLR or CRP cut-off points for the association with COVID-19 mortality (21) or the D-dimer cut-off point for the exclusion of venous thromboembolism (22). *P ≤ 0.0117; **P = 0.0077; ns *P* > 0.0500.

As only patients with severe disease were hospitalized, only those patients underwent para-clinical evaluations that included hematological studies. The total and differential leukocyte counts, the NLR, and CRP and D-dimer values were outside reference values in all patients (Fig. 4 and 5). However, deceased patients presented higher total leukocyte and neutrophil counts than recovered patients (Fig. 4). Deceased patients also exhibited more severe lymphocytopenia and eosinopenia than recovered patients (Fig. 4). Thus, the NLR was higher in deceased patients than in recovered patients (Fig. 5). There was no significant difference between recovered and deceased patients with respect to monocyte and basophil counts nor between CRP and D-dimer values (Fig. 4 and 5).

## DISCUSSION

We evaluated rNAbs against SARS-CoV-2 and simultaneously evaluated the levels of 14 sICPs: sCD28, s4-1BB, sGITR, sCD27, sHVEM, sCD80, sCTLA4, sIDO, sBTLA, sLAG-3, sTIM-3, sPD-1, sPD-L1, and sPDL2. The first five have co-stimulatory functions, and the remainder have a co-inhibitory function—primarily on T cells.

We observed that, regardless of the degree of disease severity, the three groups of COVID-19 patients presented with similarly high levels of relative neutralization, which suggests severe infection is not associated with a defective humoral immune response (23). Overall, the serum levels of sIDO, sBTLA, and sLAG-3 were significantly higher in the two groups of severe patients compared with the control group. Thus, inhibitory sICPs were not differentially upregulated according to disease severity. However, the data indicate that overall SARS-CoV-2 infection increases these sICPs in the peripheral blood. Levels of sCD27, sHVEM, and sTIM-3 were significantly higher in patients with severe disease than in controls and patients with moderate disease. Moreover, sPD-1 and sPD-L1 levels significantly differed between patients with moderate disease versus recovered patients with severe disease, while sPD-L2 was only significant between the two groups with severe disease.

Our findings on sICP levels in COVID-19 patients differ from those reported by references (10). In that study, the authors evaluated the same 14 sICPs in 109 COVID-19 patients classified as asymptomatic, mild, moderate, severe, and critical, with the same age range as in our study (20–88 years). They found that the serum levels of 11 sICPs (sGITR, s4-1BB, sTIM-3, sCD27, sLAG-3, sPD-1, sCD28, sCTLA-4, sBTLA, sHVEM, and sCD80) were persistently higher in patients with severe disease compared to moderate cases. Notably, they reported that increased levels of sIDO, s4-1BB, sTIM3, and sCD27 were related to higher disease severity. In our study, only the serum levels of sCD27, sHVEM, sTIM3, and sPD-1were higher in severe cases than moderate ones. In contrast, there was an increase in sPDL-1 values in severe cases compared to moderate ones and there was a significant increase in sPDL-2 values in the deceased group compared to the recovered group. However, a deceased group was not included in the Kong et al. study. Moreover, their patients were evaluated between January and March 2020 and were likely infected with an ancestral variant of SARS-CoV-2. In our study, we included vaccinated patients against SARS-CoV-2, and patients were infected with the delta GK/478 AY variant, which could account for these differences.

Emerging evidence has indicated that, for COVID-19, T cell-mediated immunity is generally a more reliable correlate of vaccine protection than antibody titers (24); hence, vaccination likely affected the results of sICPs values. Since sICPs are important regulators of T cell function, the quantification of sICPs could be studied as an easily determined indicator that could be considered a correlate of protection by vaccination.

Lee et al. (11) evaluated and compared 17 sICPs in 23 survivors and 15 deceased COVID-19 patients treated at an intensive care unit. They observed that the levels of sCD27, sCD40, sCTLA-4, sHVEM, and sTIM-3 were significantly higher in deceased patients than in survivors. This differs from our results, where we only observed differences in sPDL-2 values between survivors and deceased patients with severe disease. Furthermore, although we did not establish correlations between sICPs and clinical laboratory markers, as reported by Lee et al. (11), we did observe increased levels of both total and differential leukocyte counts, the NLR, CRP, and D-dimer values in patients with severe SARS-CoV-2 infection with respect to reference values in healthy subjects. Furthermore, we observed a higher NLR in deceased patients compared to survivors. Moreover, and similar to previous results by our working group (20), deceased patients had more severe lymphocytopenia and eosinopenia than recovered patients.

Some studies have found that T cell functionality decreases in patients with severe COVID-19 (25, 26), with the exhaustion related to increased expression of ICPs with co-inhibitory functions in membranous (27) and soluble forms (14). However, in this study, we also observed increased sICPs with co-stimulatory functions, such as sCD27 and sHVEM, in the sera of patients with severe COVID-19. Co-stimulatory signals initiated by the interaction between the tumor necrosis factor (TNF) ligand and the subset of the TNF receptor superfamily molecules that included CD27 and HVEM promote clonal expansion, differentiation, and the survival of antigen-primed CD4+ and CD8+ T cells (28). CD27 and its ligand CD70 are constitutively expressed on T cells, natural killer (NK) cells, and B cells (29). Additionally, given that the soluble form of CD27 is simultaneously produced in membranous form during the immune response, it is an indicator of local and systemic immune activation (30). Mouse experiments have shown that CD27 co-stimulation enhances anti-viral T cell immunity (31), while an analysis of HIV-infected patients has suggested that the CD27-CD70 interaction may maintain the CD8+ T cell response and compensate for a limited CD4+ T cell response (32). However, *in vitro* and *in vivo* studies have indicated that peripheral CD27-CD38 + B cells can promote HIV replication and disease progression (33). Still, the TNFSF14-HVEM pathway is a co-stimulatory one for T cells, whereas the BTLA-HVEM interaction inhibits T cell activation (34). Both sBTLA and sHVEM were increased in the severe COVID-19 patients included in this study, where they likely interacted and inhibited T cell activation.

PD-1 is expressed on the surface of CD4+ and CD8+ T cells, B cells, monocytes, NK cells, dendritic cells, innate lymphoid cell precursors, and mature innate lymphoid cells. The activation of PD-1 with its ligands PDL-1 and PDL-2 results in the inhibition of T cell proliferation, differentiation, cytokine secretion, and cytolytic function (35), and when ICPs are overexpressed or overactivated, immune function is inhibited. By taking advantage of this phenomenon, tumor cells that excessively activate ICPs prevent local immune cells from escaping surveillance and clearance, accelerating tumor growth. Thus, targeting the PD-1/PD-L1 signaling pathway using monoclonal antibodies has made meaningful progress in cancer immunotherapy and is currently being developed with small-molecule inhibitors (36). The soluble forms of PD-1 and PD-L1 increase the functional complexity of PD-1-PD-L1 signaling. While the exact roles of these molecules remain unknown, accumulated evidence indicates that sPD-1 and sPD-L1 may play significant roles in tumor pathogenesis (37–39). Moreover, as sPD-1 and sCTLA-4 can diffuse in serum, investigators have shown increased interest in them, given their promise in developing therapies against cancer (7).

Importantly, an experimental mouse study found that PD-1/PD-L1, but not PD-1/PD-L2, interactions regulated the severity of autoimmune encephalomyelitis (40). In another study that investigated the interaction between T cells and macrophages in an *in vitro* mouse co-culture model, the authors found that antagonizing PD-1 ligands and PDL-2, but not PDL-1, contributed to T cell and macrophage contact duration, suggesting that PDL-2 plays a more central role in the interaction between T cells and macrophages and that PD-1 ligands play different kinetic roles in T cells (41). Regarding COVID-19, we observed increased levels of sPD-1 (95% CI: Moderate = 128.8 and Rec-S = 320.7) and sPDL-1 (95% CI: Moderate = −0.1853, Rec-S = 0.7984, and Rec-D = 0.8332) in the peripheral blood of severe cases. To date, this is the first study we are aware of that demonstrates increased values of sPDL-2 (95% CI: Rec-S = 4.959 and Rec-D = 7.858) in patients with severe disease that has led to death.

Among the main limitations of our study were the small sample size and the lack of paired samples to evaluate the changes in serum biomarkers over the course of the disease. These limitations could have led to not observing a larger difference in sPDL-2 levels between the COVID-19 groups.

In conclusion, this study demonstrated that sCD27, sHVEM, sTIM-3, sPD-1, and sPDL-1 levels were significantly higher in patients with severe COVID-19 than those with moderate disease. Our results suggest that PDL-2 should be examined as a potential biomarker of fatality in COVID-19 patients.

## MATERIALS AND METHODS

### Design

This was a cross-sectional, retrospective cohort study.

### Setting

Individuals testing positive to SARS-CoV-2 on a rapid antigen test (outpatients) or a PCR test (hospitalized patients) at the Mexican Social Security Institute (IMSS) in Morelia, Michoacán, Mexico, between September 2021 and February 2022 and whose clinical data were registered in the Online Notification System for Epidemiological Surveillance (SINOLAVE) database were included in the study. Outpatients with mild or moderate COVID-19 symptoms seen at the Respiratory Care module of Social Security at the IMSS clinic No. 80 were included. These outpatients provided a peripheral blood sample (BS) for our work team. Serum samples from hospitalized patients with severe COVID-19 were donated by the Zone General Hospital No. 83 clinical laboratory, which was converted into a COVID-19 hospital during the pandemic. Peripheral BSs from hospitalized patients were obtained during routine clinical laboratory tests. Patients for whom there was a BS, but their clinical data were not found in the SINOLAVE database, were eliminated from the study. Serum from SARS-CoV-2 negative subjects was obtained before the start of the pandemic.

### Subjects

In Mexico, on 24 December 2020, the first stage of administering vaccines against COVID-19 began (42). This resulted in a significant decrease in confirmed cases, hospital occupancy, and deaths from COVID-19. By September 2021, when our clinical study began, approximately 71% of adults aged 18 years and older had already completed their vaccination schedule, while the remaining 29% had received the first dose or none at all (43). This accounts for why we worked with a small sample size.

Patients were categorized into a SARS-Cov-2 negative control group (*n* = 16) and three groups of SARS-CoV-2 positive patients. These groups included patients with mild or moderate disease (*n* = 16), recovered patients with severe disease (*n* = 13), and deceased patients with severe disease (*n* = 16). The diagnosis of severity of the COVID-19 patients included in the SINOLAVE database was established by medical review according to the clinical guide for the treatment of COVID-19 in Mexico (44).

### Blood samples

Venous BSs were collected in BD Vacutainer tubes. Aliquots of 200 µL of serum were obtained and frozen at −20°C for subsequent analysis.

### Clinical laboratory information

White blood cell (WBC) analyses of patients with severe disease were performed using an automatic hematology analyzer from Sysmex XN-2000TM (Kobe, Japan). CRP and D-dimer values were determined using the latex agglutination test in serum (Kabla Comercial SA de CV, Monterrey, Mexico). The NLR was obtained by dividing the neutrophil count by the lymphocyte count. A comparison of paired samples was performed on the total and differential leukocyte counts, the NLR, CRP, and D-dimer values. Total and differential leukocyte counts were compared using 42 BSs from recovered patients and 30 BSs from deceased patients. The NLR was compared on 42 BSs from recovered patients and 30 BSs from deceased patients. The CRP was compared on 18 BSs from recovered patients and 32 BSs from deceased patients, and D-dimer values were compared on 58 BSs from recovered patients and 56 BSs from deceased patients.

### Demographic and clinical data

Patient demographic characteristics, clinical data, the variant of SARS-CoV-2 (obtained via sequencing), and the classification of COVID-19 patients according to disease severity were obtained from the SINOLAVE database.

### Seroconversion to SARS-CoV-2

The detection of IgG antibodies against the S1 subunit of the SARS-CoV-2-spike protein in serum was conducted using a qualitative immunoassay with the anti-SARS-CoV-2 ELISA IgG EUROIMMUN kit, Order No: EI 2606-9601 G (PekinElmer company, Seekamp, DE), following the manufacturer’s instructions. Briefly, 100 µL of the calibrator, positive samples, and negative controls were loaded into individual microplate wells, covered with protective foil, and incubated for 60 min at 37°C. The wells were washed 3× using 300 µL of wash buffer. Next, 100 µL of peroxidase-labeled anti-human IgG was added to each microplate well and incubated for 30 min at 37°C. The wells were then washed 3× as described above, and 100 µL of chromogen/substrate solution was added to each well. The microplate was then incubated for 30 min at room temperature and protected from direct sunlight. Next, 100 µL of stop solution was added to each well, and photometric evaluations were made at 450 nm. Unit values (RU) were obtained using the following equation:


RU=(OD450 of the sample×unit of the calibrator:10 RU/mL)/OD450 of the calibrator

Values greater than 11 RU/mL were considered positive for IgG antibodies against the SARS-CoV-2-spike protein.

### Determination of the rNAbs in sera (% inhibition RBD-ACE2)

Determining the rNAbs was conducted with an ELISA using the cPassTM SARS-CoV-2 Neutralization Antibody Detection kit (RUO), REF: L00847 (GenScript biotech corporation, Piscataway, NJ, USA), a SARS-CoV-2 surrogate virus neutralization test based on antibody-mediated blockage of ACE2–spike protein-protein interaction that does not require the use of the virus, following the manufacturer’s instructions. First, 100 µL of the samples and controls, previously diluted to 1:10 with dilution buffer, were pre-incubated with 100 µL of RBD-HRP (1:1,000 dilution) to allow for the interaction and binding of neutralization antibodies. The mixture was incubated at 37°C for 30 min, added to a capture plate pre-coated with the human ACE2 protein, and incubated at 37°C for 15 min. The neutralization antibody/RBD-HRP complex was removed by washing the plate 4× with 260 µL of wash solution per well. Then, 100 µL of TMB solution was added to each well and incubated in the dark at 25°C for 15 min, which gave it a blue color. After adding 50 µL of stop solution, the reaction was stopped, and the color turned yellow. The absorbance was read immediately in a microtiter plate reader at 450 nm. The test was calibrated for semi-quantitative detection using a calibration curve with three dilutions (1:20, 1:60, 1:80) in duplicate of the positive and negative controls. The rNAbs (% inhibition RBD-ACE2) were obtained by the following equation:


% inhibition RBD-ACE2=1−OD of the positive control/OD of the negative control×100

The absorbance of the sample was inversely dependent on the titer of the anti-SARS-CoV-2 neutralizing antibodies.

### Performance of quantitative, multiplexed sICP measurements

The ProcartaPlexTM Human Immuno-Oncology Checkpoint Marker Panel 1 14-Plex Immunoassay kit, lot number: 308037-011 (Thermo-Fisher Scientific, Waltham, MA, USA) was used for the simultaneous detection and quantification of 14 analytes [sBTLA, sCD27, sCD28, sCD80, sCD137 (s4-1BB), sCD152 (sCTLA4), sGITR, sHVEM, sIDO, sLAG-3, sPD-1, sPD-L1, sPDL2, and sTIM-3] from sera using magnetic bead technology from the LuminexTM 200TM platform (Thermo Fisher Scientific). The plate map for standard samples and blanks was previously defined. Seven antigen standards using a fourfold serial dilution of the reconstituted standard were added to the individual PCR 8-tube strip. An assay buffer was used for the background and was added to the eight tubes. Each bead vial was vortexed for 3 s, and 50 µL of magnetic beads was added to individual microplate wells. Magnetic beads were washed 2× using a hand-held magnetic plate following the washing protocol suggested by the manufacturer. Then, 25 µL of standard, universal assay buffer or sample was added to the beads. The plate was sealed and incubated with shaking at room temperature for 60 min and then incubated overnight without shaking at 4°C. On day 2, the magnetic beads were washed 2×, and 50 µL of streptavidin-PE was added to the individual microplate wells. The plate was sealed and incubated with shaking at room temperature for 30 min. Immediately afterward, beads were washed 2× and resuspended by adding 120 µL of reading buffer. The plate was sealed and incubated with shaking at room temperature for 5 min. Data were acquired using a LuminexTM 200 system and the ProcartaPlexTM analysis application: https://apps.thermofisher.com/apps/procartaplex.

### Statistical analysis

Data analyses and graphing were conducted using GraphPad Prism software version 9.0 (GraphPad Software Inc., Boston, MA, USA). Differences between groups according to relative neutralizing values, sICPs levels, leukocyte values, and D-dimer and CRP values were analyzed using the Kruskal-Wallis test with Dunnett’s multiple comparison, the paired *t*-test, or the paired Wilcoxon signed-rank test. Categorical variables were analyzed using the Chi-squared test or Fisher’s exact test. A *P*-value < 0.05 was considered statistically significant.
